# Benchmarking Analysis of the Accuracy of Classification Methods Related to Entropy

**DOI:** 10.3390/e23070850

**Published:** 2021-07-01

**Authors:** Yolanda Orenes, Alejandro Rabasa, Jesus Javier Rodriguez-Sala, Joaquin Sanchez-Soriano

**Affiliations:** I.U.I. Centro de Investigación Operativa (CIO), Universidad Miguel Hernandez de Elche, 03202 Elche, Spain; yolanda.orenes@alu.umh.es (Y.O.); a.rabasa@umh.es (A.R.); jesuja.rodriguez@umh.es (J.J.R.-S.)

**Keywords:** entropy, classification methods, intuitive classification method, performance measures, benchmarking

## Abstract

In the machine learning literature we can find numerous methods to solve classification problems. We propose two new performance measures to analyze such methods. These measures are defined by using the concept of proportional reduction of classification error with respect to three benchmark classifiers, the random and two intuitive classifiers which are based on how a non-expert person could realize classification simply by applying a frequentist approach. We show that these three simple methods are closely related to different aspects of the entropy of the dataset. Therefore, these measures account somewhat for entropy in the dataset when evaluating the performance of classifiers. This allows us to measure the improvement in the classification results compared to simple methods, and at the same time how entropy affects classification capacity. To illustrate how these new performance measures can be used to analyze classifiers taking into account the entropy of the dataset, we carry out an intensive experiment in which we use the well-known J48 algorithm, and a UCI repository dataset on which we have previously selected a subset of the most relevant attributes. Then we carry out an extensive experiment in which we consider four heuristic classifiers, and 11 datasets.

## 1. Introduction

Classification is one of the most relevant topics in machine learning [1,2,3,4]. In general, the purpose of supervised classification is to predict the correct *class*, among a set of known *classes*, of a new observation given, based on the knowledge provided by a dataset, known as “*training data*”. In addition, the classification problem is very important in decision-making in many different fields, so it is not difficult to find applications in fields such as medicine, biotechnology, marketing, security in communication networks, robotics, image and text recognition... Three issues in classification problems are the attribute subset selection, the design and implementation of classifiers, and the performance evaluation of classifiers [1,2,3,4]. In this paper, we will focus mainly on the latter.

On the other hand, entropy appears in statistics or information theory as a measure of diversity, uncertainty, randomness or even complexity. For this reason, we can find the use of entropy in the feature selection problem and the design of classifiers. Shannon [5] introduced entropy in the context of communication and information theory. This concept has been used frequently in information-based learning models [2]. Two extensions of the Shannon entropy measure, which are also frequently used, are the Renyi’s entropy [6] and the Tsallis’ entropy [7]. In [8], a review on generalized entropies can be found.

One of the most frequent difficulties found in the analysis of a dataset is that of high dimensionality, since when there are too many variables the analysis is more difficult and computationally expensive, there may be correlated variables, redundant variables or even noisy variables. All of these problems can lead to poorer performance of classifiers. Thus, to solve these difficulties, one of two alternatives is commonly used: (1) reducing the dimension by transforming data, or (2) selecting a subset of characteristics while keeping most of the information in the dataset; this approach is known as feature selection. For example, in [9] the linear discriminant analysis and the RBS feature selection method are compared. An advantage of the feature selection approach is that the original meaning of the variables is kept. In classification problems, where there is a nominal target variable (the consequent), the selection of the most relevant variables is not a trivial matter. The issue of feature selection has already been addressed in many studies in the field of machine learning by using different approaches including information entropy [10,11,12,13,14,15,16,17,18,19,20,21,22,23,24,25,26,27,28,29,30,31,32,33,34]. Liu and Yu [35] reviewed feature selection algorithms for classification and clustering, and categorize them to facilitate the choice of the most suitable algorithm for the analysis of a particular dataset.

Many of the feature selection procedures incorporate the use of their own classifier to measure the quality of the selection, therefore, on many occasions it is possible to identify the feature selection method with the classifier itself, as can happen in wrapper and embedded methods of feature selection. There are different types of classification algorithms depending on its structure or the principles behind them. Thus, we can find classification algorithms (1) based on induction of decision tree algorithms such as ID3 [36] and its extension C4.5 [37], the classification and regression tree algorithm CART [38], and their extensions to random forest algorithms [39,40,41]; (2) based on similarities such as K-nearest neighbor algorithms [42,43] and their extensions to instance-based algorithms such as IBL [44]; (3) based on separation methods in vector spaces such as support vector machine algorithms [45,46]; or (4) based on probabilistic or statistical concepts and methods such as linear discriminant analysis [47], logistic regression or naïve Bayes algorithms [48,49]; among others. For details on classification and learning problems and their algorithms see [1]. Moreover, we can find in the machine learning literature many papers in which different concepts and methods from information entropy are used together with learning classification algorithms to design new classifiers to be applied in different contexts [50,51,52,53,54,55,56,57,58,59,60].

Given the same dataset, not all classifiers are equally accurate in their predictions. The accuracy achieved by a classification model depends on several factors such as the algorithm’s own implementation, the heuristics of pruning and built-in boosting, the dataset used, and even the set of variables finally chosen for the construction of the model. Therefore, the analysis of the performance of classifiers is relevant in order to determine which works better. It is known that there is a lower bound on the error rate that can be achieved by classifiers: the Bayes error [61]. This error is associated with the Bayes classifier, which assigns an observation to the class with the highest posterior probability [61]. Therefore, this classifier and its associated error can be considered as benchmarks to evaluate the performance of a given classifier. However, the Bayes error can be computed only for a few number of problems. Therefore, different approximations and bounds of this error can be found in the literature (see, for example, Kumer and Ghosh [62] and the references herein). In the machine learning literature, there are different measures of the performance of a classifier and we can find various works that analyze the performance of different classifiers according to them. Costa et al. [63] showed that the most usual evaluation measures in practice were inadequate for hierarchical classifiers and reviewed the main evaluation measures for hierarchical classifiers. Sokolova and Lapalme [64] analyzed how different types of changes in the confusion matrix affected performance measures of classifiers. In particular, they studied the invariance properties of 24 performance measures for binary, multi-class, multi-labeled and hierarchical classifiers. Ferri et al. [65] carried out a experiment to analyze 18 different performance measures of classifiers. They also studied the relationships between the measures and their sensitivity from different approaches. Parker [66] analyzed the incoherences of seven performance measures for binary classifiers from both a theoretical and an empirical point of view in order to determine which measures were better. Labatut and Cherifi [67] studied properties and the behavior of 12 performance measures for flat multi-class classifiers. Jiao and Du [68] reviewed the most common performance measures used in bioinformatics predictors for classifications. Valverde-Albacete and Peláez-Moreno [69,70,71,72] analyzed classification performance with information-theoretic methods. In particular, they proposed to analyze classifiers by means of entropic measures on their confusion matrices. To do this, they used the de Finetti entropy diagram or entropy triangle and a suitable decomposition of a Shannon-type entropy, and then defined two performance measures for classifiers: the entropy-modified accuracy (EMA) and the normalized information transfer (NIT) factor. The EMA is the expected proportion of times the classifier will guess the output class correctly, and the NIT factor is the proportion of available information transferred from input to output. The quotient of these two measures provides information on how much information is available for learning.

In this paper, we focus on the definition of performance measures. In particular, following the ideas on agreement coefficients from statistics, the Cohen’s κ [73] and the Scott’s π [74], which have also been used as performance measures of classifiers [75], we consider three performance measures closely related to them. Those statistics were originally defined to measure the concordance level between the classifications made by two evaluators. The mathematical formula is the following:

(1) $$\mathrm{Concordance}\;\mathrm{level}=\frac{P_{0}-P_{e}}{1-P_{e}},$$

where $P_{0}$ represents the observed proportion of classifications on which the two evaluators agree when classifying the same data independently; and $P_{e}$ is the proportion of agreement to be expected on the basis of chance. Depending on how $P_{e}$ is defined the Cohen’s κ or the Scott’s π are obtained. In machine learning, these statistics are used as performance measures by considering the classifier to be evaluated and a random classifier, where $P_{0}$ is the accuracy of the classifier. In this paper, we look at these performance measures from another point of view and define two new performance measures based on the Scott’s π. In particular, we use the interpretation given in Goodman and Kruskal [76] for the λ statistics. Thus, we consider three benchmark classifiers, the random classifier and two intuitive classifiers. The three classifiers assign classes to new observations by using the information of the frequency distribution of all attributes in the training data. To be more specific, the random classifier, X, predicts by random with the frequency distribution of the classes at hand, while the first intuitive classifier, V, predicts the most likely outcome for each possible observation with the frequency distribution of the classes in the training data, and the second intuitive classifier, I, predicts the most likely outcome for each possible observation with the joint frequency distribution of all attributes in the training data. The two described intuitive classifiers were postulated, built, and analyzed but rejected in favor of more modern classifier technologies before 2000. However, they could still be useful to define other performance measures in the style of the Cohen’s κ or the Scott’s π. Thus, in order to evaluate a classifier we determine the proportional reduction of classification error when we use the classifier to be evaluated with respect to using one of the benchmark classifiers. In this sense, $P_{0}$ is the accuracy of the classifier to be evaluated and $P_{e}$ is the (expected) accuracy of the benchmark classifier. In the case where the benchmark classifier is the random classifier we obtain a performance measure like the Scott’s π, but the interpretation given is different from the usual one in the machine learning literature. This is also an interesting approach of performance evaluation of classifiers because we can measure how advantageous a new classifier is with respect to three simple benchmark classifiers which can be seen as the best common sense options for non-expert (but sufficiently intelligent and with common sense) people, and whose error rates are simpler to determine than the Bayes error.

On the other hand, we analyze the relationship between the three benchmark classifiers and different aspects of the entropy of the dataset. Thus, the random classifier X and the intuitive classifier V are directly related to the entropy of the target attribute, while the intuitive classifier I is closely related to the entropy of the target attribute when all dataset is considered, i.e., to the conditional entropy of the target attribute given the remaining variables in the dataset. With this relationships in mind, we can analyze the performance of classifiers taking into account the entropy of the dataset [77]. This is an interesting approach because it allows us to identify under what conditions of information uncertainty (measured by means of entropy) a classifier works better.

To the best of our knowledge, the main contributions of the paper to the machine learning literature are the following:

1. We consider the random classifier and two intuitive classifiers as benchmark classifiers. These classifiers can be considered as simple, intuitive and natural for common sense non-expert decision-makers.
2. We define three new performance measures of classifiers based on the Scott’s π, the accuracy of classifiers, and the benchmark classifiers.
3. We interpret our performance measures of classifiers in terms of proportional reduction of classification error. Therefore, we measure how much a classifier improves the classification made by the benchmark classifiers. This interpretation is interesting because it is easy to understand and, at the same time, we determine the gain in accuracy related to three simple classifiers. In a sense, they provide information on whether the design of the classifier has been worth the effort.
4. The three performance measures of classifiers lie in the interval [−1,1], where −1 means that the classifier in evaluation worsens by 100% the correct classification made by the corresponding benchmark classifier, this corresponds to the classifier assigns incorrectly all observations, and 1 means that the classifier reduces by 100% the incorrect classification made by the corresponding benchmark classifier, this corresponds to the classifier assigns correctly all observations.
5. The benchmark classifiers catch the entropy of the dataset. The random classifier X and the intuitive classifier V measure the entropy of the target attribute, and the intuitive classifier I reflects the conditional entropy of the target attribute given the remaining variables in the dataset. Therefore, they allow us to analyze the performance of a classifier taking into account the entropy in the dataset. These measures, particularly that based on the intuitive classifiers, offer different information than other performance measures of the classifiers, which we consider to be interesting. The aim, therefore, is not to substitute for any known performance measure, but to provide a measure of a different aspect of the performance of a classifier.
6. We carry out an intensive experiment to illustrate how the proposed performance measures works and how the entropy can affect the performance of a classifier. For that we consider a particular dataset and the classification algorithm J48 [78,79,80], an implementation provided by Weka [75,81,82,83], of the classic C4.5 algorithm presented by Quinlan [36,37].
7. In order to validate what was observed in the previous experiment, we carried out an extensive experiment using four classifiers implemented in Weka and 11 datasets.

The rest of the paper is organized as follows. In Section 2, we provide the methodology and materials used in the paper. In particular, the method of feature selection, the algorithm of the intuitive classifier I, the description of several heuristic classifiers implemented in Weka [75,81,82,83], and the definition and theoretical analysis of the performance measures introduced in this paper. In Section 3, we carry out the experiment to illustrate how the performance measures work and how they can be used to analyze the classifiers’ performance in terms of entropy. In Section 4, we discuss the results obtained and conclude. Tables are included in Appendix A.

## 2. Materials and Methods

### 2.1. Method and Software Used for Feature Selection

The method used to perform the selection and ranking of the most influential variables is Gain Ratio Attribute Evaluation [25] (implemented in Weka [75,81,82,83]). This measure, GR(att) on Equation (2), provides an objective criterion for sorting explanatory variables by importance versus the target variable. Gain Ratio by its own design penalizes the proliferation of nodes and meliorates the variables that are distributed so uniformly. The gain ratio of each attribute is calculated using the following formula:

(2) $$GR\left(att\right)=\frac{IG(att)}{H(att)},$$

where (*IG*) is a measure to evaluate the informational gain provided by each attribute, which is considered to be a popular measure to evaluate attributes. In particular, it is the difference between the entropy of the consequent attribute and the entropy when *att* is known, H(att). Thus, the feature selection method calculates the informational gain for each attribute *att* [25].

### 2.2. Methodology and Software for the Intuitive Classification Method I

**Figure 1:**
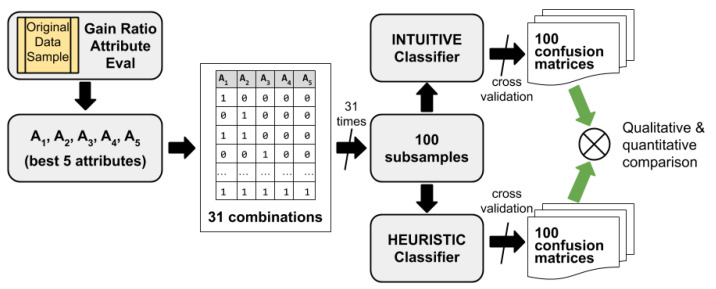
Experiment for each data scenario.

The basic idea of the intuitive classifier I is to generate classification rules from a dataset where all values are discrete (text tags). Dataset data will have *C* columns or attributes $\left(A_{1},\ldots ,A_{C}\right)$. One of the attributes ($A_{C}$ in the Figure 1) is the target variable, used to classify instances. The remaining attributes $\left(A_{1},\ldots ,A_{C-1}\right)$ are the explanatory variables of the problem or antecedents.

(3) $$rule:\underset{\mathrm{left}\;\mathrm{side}}{\underset{︸}{<A_{1}=V_{1}>,\ldots ,<A_{C-1}=V_{C-1}>}}\to \underset{\mathrm{right}\;\mathrm{side}}{\underset{︸}{<A_{C}=V_{C}>}}$$

A classification rule will consist of an antecedent (left side of the rule) and a consequent (right side of the rule), as illustrated in Equation (3). The antecedent will be composed of C−1 attribute/value pairs ($<A_{i}=V_{i}>$), where attributes are the explanatory variables. The consequent will consist of an attribute pair (target variable/value) in the form $<A_{C}=V_{C}>$.

The intuitive classifier I counts the more repeated values within the data sample. In our opinion this could be what any non-expert person would do to try to identify the most likely patterns of a data sample by applying common sense. The algorithm of the intuitive classifier I (see Algorithm 1) performs a scan comprehensive by all records in the dataset and counts how many times each combination of values is given in the left side of the rule (antecedent), to that amount of what we will call *rule support* (R. supp). Analogously, given an antecedent, for each classification rule, the algorithm counts the number of times each of the its possible consequences or right part of the rule. We call it *rule confidence* (R. conf). (see Algorithm 1).
**Algorithm 1** Pseudo-code of the algorithm of the intuitive classifier I.1:**INPUT**:2:*S*: training data sample with *C* columns and *N* rows3:C−1 attributes are the antecedent4:1 class variable is the consequent5:START ALGORITHM6:CRS ←⌀ {/*initialized as void set*/}7:**for** each row in *S* **do**8: **if** there exists a rule Rj in CRS such that Antecedent(Rj) = Antecedent(row) **and** Consequent(Rj) = Consequent(row) **then**9:  **for all** Ri in CRS such that Antecedent(Ri) = Antecedent(row) **do**10:   Ri.supp ← Ri.supp + 111:  **end for**12:  Rj.conf ← Rj.conf + 113: **else**14:  R ← New Rule15:  R.antecedent ← Antecedent(row)16:  R.consequent ← Consequent(row)17:  R.supp ← 118:  R.conf ← 119:  **for all** Ri in CRS such that Antecedent(Ri) = Antecedent(row) **do**20:   Ri.supp ← Ri.supp + 121:  **end for**22:  CRS← CRS + R {/*add R to CRS*/}23: **end if**24:**end for**25:**return**CRS: Classification Rule Set {/*OUTPUT*/}26:END ALGORITHM

Note that each rule (R) of the set of rules (CRS), generated according to Algorithm 1, has associated both support and confidence values (R. supp, R. conf). These values are, as indicated above, the number of times the antecedent is repeated in the sample of data and, the number of times that, given a particular antecedent, its class of the consequent is repeated in the data sample. These two counters allow us to determine which patterns are the most repeated. This model, formed by the whole of CRS rules, predicts the class variable of an instance “s” by applying Algorithm 2.

Algorithm 2 infers the value of instance class “s”, using the set rule CRS whose antecedent most closely resembles the antecedent of “s” (matching a greater number of attributes). In the case where there are multiple rules with the same number of matches, that which has a larger support is selected. If there are several rules with equal support, the most trusted is chosen. Once that rule is identified, the predicted class is the value of the consequent of the selected rule.
**Algorithm 2** Pseudo-code of the algorithm to predict with a CRS model.1:**INPUT**:2:*s*: test row with C−1 antecedent attributes3:CRS: Classification Rule Set4:**USE**: RSS: Rule subset5:START ALGORITHM6:**for**c=C−1**to** 1 **do**7: RSS ← {Ri ∈ CRS / c attributes of s are equal to c attributes of Ri}8: **if** RSS ≠⌀ **then**9:  R ← R1 {/* R1 is the first rule of RSS */}10:  **for** j=2 **to** |RSS| **do**11:   **if** R.supp < Rj.supp **then**12:    R ← Rj13:   **else if** R.supp = Rj.supp **and** R.conf < Rj.conf **then**14:    R ← Rj15:   **end if**16:   **return** R.consequent17:  **end for**18: **end if**19:**end for**20:**return** The resulting predicted class for row s (the consequent of a rule of CRS) {/*OUTPUT*/}21:END ALGORITHM

### 2.3. Methodology and Software for the Heuristic Classifiers

For the generation of predictive models from the heuristic approach, we consider several heuristic classifiers: J48, Naïve Bayes, SMO, and Random Forest.

The decision tree learner J48 [78,79,80] is an implementation provided by Weka of the classic C4.5 algorithm [36,37]. J48 extends some of the functionalities of C4.5 such as allowing the post-pruning process of the tree to be carried out by a method based on error reduction or that the divisions over discrete variables are always binary, among others [75]. These decision trees are considered supervised classification methods. There is a dependent or class variable (variable of a discrete nature), and the classifier, from a training sample, determines the value of that class for new cases. The tree construction process begins with the root node, which has all training examples or cases associated. First, the variable or attribute from which to divide the original training sample (root node) is chosen, seeking that in the generated subsets there is minimal variability with respect to the class. This process is recursive, i.e., once the variable with the highest homogeneity is obtained with respect to the class in the child nodes, the analysis is performed again for each of the child nodes. This recursive process stops when all leaf nodes contain cases of the same class, and then over-adjustment should be avoided, for which the methods of pre-pruning and post-pruning of trees are implemented.

We also consider the Naïve Bayes algorithm implemented in Weka [75,81,82,83] which is a well-known classifier [48,49] based on the Bayes Theorem. Details on Naïve Bayes classifiers can be found almost in any data science or machine learning book. On the other hand, Ref. [81] is an excellent reference for the Weka software.

The SMO is an implementation in Weka [75,81,82,83] of the Platt’s sequential minimal optimization algorithm [84,85,86] for training a support vector machine classifier [45]. SMO is a simple algorithm to quickly solve the support vector machine quadratic problems by means of the decomposition of the overall quadratic problem into smaller quadratic sub-problems which are easier and faster to be solved.

Finally, we will also use the random forest classifier implemented in the Weka software [75,81,82,83]. Random forests classifiers [41] consist of ensembles of decision trees which are built from randomly selected subset of training set, and the final classification is the result of the aggregation of the classification provided by each tree.

### 2.4. Evaluation Measures

The evaluation of classifiers or models to predict is very important because it allows us (1) to compare different classifiers or models to make the best choice, (2) to estimate how the classifier or model will perform in practice, and (3) to convince the decision maker that the classifier or model will be suitable for its purpose (see [1,2]). The simplest way to evaluate a classifier for a particular problem given by a dataset is to consider the ratio of correct classification. If we denote by Z the classifier and by D the dataset, then the performance of Z classifying a particular attribute (the consequent) in D is given by

(4) $$acc\left(Z\left(D\right)\right)=\frac{\mathrm{number}\mathrm{of}\mathrm{correct}\mathrm{predictions}}{\mathrm{total}\mathrm{predictions}}.$$

This measure is known as *accuracy*. There are other evaluation measures [1,2], but we focus in this paper on defining new measures based in some way on the concepts of proportional reduction of the classification error [76] and entropy [5].

Our approach for defining evaluation measures based on entropy is by considering simple classifiers that capture the entropy of the problem. These classifiers play the role of benchmark when evaluating other classifiers.

Let us consider a dataset D with *N* instances (rows) and *C* attributes (columns) such that attributes $A_{1},A_{2},\ldots ,A_{C-1}$ are considered the explanatory variables (antecedents) and $A_{C}$ is the attribute to be explained (consequent) or predicted. Let $a_{C1},a_{C2},\ldots ,a_{CK}$ be the categories or classes of variable $A_{C}$, and let $p_{C1},p_{C2},\ldots ,p_{CK}$ be the relative frequencies of those categories in D. Associated with this problem, we can consider a random variable *X* from the sample space $\Omega =\left\{a_{C1},a_{C2},\ldots ,a_{CK}\right\}$ to R, such that $X(a_{Cj})=j$, and $Prob\left(X=j\right)=p_{Cj}$. Therefore *X* has the non-uniform discrete distribution $D(p_{C1},p_{C2},\ldots ,p_{CK})$, i.e., $X∼D(p_{C1},p_{C2},\ldots ,p_{CK})$. This *X* can be considered the *random classifier* for the consequent $A_{C}$ in the dataset D, defined as

(5) $$X\left(A_{C},D\right)\left(i\right)=X\left(i\right),$$

where *i* is an observation or instance. Furthermore, we can define another simple and intuitive classifier for the consequent $A_{C}$ in the dataset D as follows

(6) $$V\left(A_{C},D\right)\left(i\right)=\arg\max\left\{p_{C1},p_{C2},\ldots ,p_{CK}\right\},$$

where *i* is an observation or instance, i.e., this intuitive classifier predicts the most likely outcome for each possible observation with the frequency distribution of the consequent $A_{C}$.

If we take the *N* instances of the dataset, then the classification of each instance *i* by the random classifier *X* has a categorical, generalized Bernoulli or multinoulli distribution with parameter $p_{i}$, where $p_{i}$ is the frequency associated with the category that attribute $A_{C}$ takes for the instance *i*, i.e., $X\left(i\right)∼B\left(p_{i}\right)$. Therefore, the expected number of success in the classification of the *N* instances is given by

(7) $$E\left(\sum_{i=1}^{N}X\left(i\right)\right)=\sum_{i=1}^{N}E\left(X\left(i\right)\right)=\sum_{i=1}^{N}p_{i}=\sum_{j=1}^{K}p_{Cj}Np_{Cj}=N\sum_{j=1}^{K}p_{Cj}^{2}.$$

Assuming that the classification of each instance is made independently, the variance of the number of success in the classification of the *N* instances is given by

(8) $$V\left(\sum_{i=1}^{N}X\left(i\right)\right)=\sum_{i=1}^{N}V\left(X\left(i\right)\right)=\sum_{i=1}^{N}p_{i}\left(1-p_{i}\right)=\sum_{j=1}^{K}p_{Cj}Np_{Cj}\left(1-p_{Cj}\right)=N\sum_{j=1}^{K}p_{Cj}^{2}\left(1-p_{Cj}\right).$$

Note that if we consider a set of instances different from dataset D then Equations (7) and (8) would be given by

(9) $$E\left(\sum_{i=1}^{N^{\prime }}X\left(i\right)\right)=\sum_{j=1}^{K}N_{Cj}^{\prime }p_{Cj}\;\;\mathrm{and}\;\;V\left(\sum_{i=1}^{N^{\prime }}X\left(i\right)\right)=\sum_{j=1}^{K}N_{Cj}^{\prime }p_{Cj}\left(1-p_{Cj}\right),$$

where $N_{Cj}^{\prime }$ is the number of instances for which attribute $A_{C}$ takes the value $a_{Cj}$.

Likewise, if we are interested in the ratio of success in the classification, then Equation (7) simply becomes

(10) $$E\left(\sum_{i=1}^{N}X\left(i\right)\right)=\sum_{j=1}^{K}p_{Cj}^{2}.$$

Thus, Equation (10) provides the expected accuracy of the random classifier X, i.e.,

(11) $$E\left(\sum_{i=1}^{N}X\left(i\right)\right)=E\left(acc(X\left(A_{C},D\right))\right).$$

In the same way, we can arrive at the accuracy of the classifier V is

(12) $$acc\left(V\left(A_{C},D\right)\right)=\max\left\{p_{C1},p_{C2},\ldots ,p_{CK}\right\}.$$

On the other hand, the Shannon entropy [5] of attribute $A_{C}$ in dataset D is given by

(13) $$H^{S}\left(A_{C},D\right)=-\sum_{j=1}^{K}p_{Cj}\log_{2}p_{Cj}.$$

Shannon entropy can be seen as a Renyi’s entropy measure [6] or a Tsallis’ entropy measure [7], which have the following mathematical expressions for attribute $A_{C}$ in dataset D,

(14) $$H^{R,\alpha }\left(A_{C},D\right)=\frac{1}{1-\alpha }\log_{2}\left(\sum_{j=1}^{K}p_{Cj}^{\alpha }\right),\;\mathrm{and}\;$$

(15) $$H^{T,\alpha }\left(A_{C},D\right)=\frac{1}{\alpha -1}\left(1-\sum_{j=1}^{K}p_{Cj}^{\alpha }\right),$$

respectively.

Renyi’s and Tsallis’ entropy measures coincide with the Shannon entropy when α goes to 1, therefore Shannon’s measure of entropy is seen as a Renyi’s entropy measure or a Tsallis’ entropy measure of order α=1. If we consider the Renyi’s entropy measure and the Tsallis’ entropy measure of order α=2, we obtain

(16) $$H^{R,2}\left(A_{C},D\right)=-\log_{2}\left(\sum_{j=1}^{K}p_{Cj}^{2}\right),\;\mathrm{and}\;$$

(17) $$H^{T,2}\left(A_{C},D\right)=\left(1-\sum_{j=1}^{K}p_{Cj}^{2}\right).$$

The entropy measures given in Equations (16) and (17) are very closely related to Equation (10), which measures the expected ratio of success in the classification of the random classifier X.

Now, we have the following result which relates the expected ratio of success of the random classifier X and the different entropy measures above of consequent $A_{C}$ when it is binary.

**Theorem** **1.** Let D, and $D^{*}$ be two datasets with the same attributes and $A_{C}$ a binary attribute which is considered the consequent. Then, the following statement holds

*1.*  $H^{S}\left(A_{C},D\right)>H^{S}\left(A_{C},D^{*}\right)\Leftrightarrow H^{R,2}\left(A_{C},D\right)>H^{R,2}\left(A_{C},D^{*}\right)$.
*2.*  $H^{S}\left(A_{C},D\right)>H^{S}\left(A_{C},D^{*}\right)\Leftrightarrow H^{T,2}\left(A_{C},D\right)>H^{T,2}\left(A_{C},D^{*}\right)$.
*3.*  $H^{S}\left(A_{C},D\right)>H^{S}\left(A_{C},D^{*}\right)\Leftrightarrow \sum_{j=1}^{2}p_{Cj}^{2}<\sum_{j=1}^{2}p_{Cj}^{*2}$.

**Proof of Theorem 1.**  In order to prove the theorem all you need is to prove statement 3, because the other two statements follow from the mathematical expressions of $H^{R,2}$, and $H^{T,2}$ and statement 3. Let $p_{C1},p_{C2}$ and $p_{C1}^{*},p_{C2}^{*}$ be two frequency distributions of $A_{C}$ such that the entropy associated with the first is greater than the entropy associated with the second. Consider that $p_{C1}\neq p_{C1}^{*}$, then $p_{C2}\neq p_{C2}^{*}$. Otherwise, the result immediately follows. Since the entropy of the first frequency distribution is greater than the entropy of the second frequency distribution, we know that $|p_{C1}-p_{C2}\left|<\right|p_{C1}^{*}-p_{C2}^{*}|$. Let us suppose without loss of generality that $p_{C1}>p_{C1}^{*}$. Since $p_{C1}+p_{C2}=p_{C1}^{*}+p_{C2}^{*}=1$, $p_{C2}<p_{C2}^{*}$.

On the other hand, we have that

(18) $$p_{C1}^{2}+p_{C2}^{2}-\left(p_{C1}^{*2}+p_{C2}^{*2}\right)=p_{C1}^{2}+{\left(1-p_{C1}\right)}^{2}-\left(p_{C1}^{*2}+{\left(1-p_{C1}^{*}\right)}^{2}\right).$$

After some calculations, we have that

(19) $$p_{C1}^{2}+p_{C2}^{2}-\left(p_{C1}^{*2}+p_{C2}^{*2}\right)=-2p_{C1}+2p_{C1}^{*}<0.$$

Therefore, $\sum_{j=1}^{2}p_{Cj}^{2}<\sum_{j=1}^{2}p_{Cj}^{*2}$.

The proof of the converse follows similarly.   □

Theorem 1 cannot be extended to attributes with more than 2 possible values, as the following example shows.

**Example** **1.** *Consider two datasets D and $D^{\prime }$, and a common attribute A for both with three possible values {a,b,c}, such that $p_{a}=0.54$, $p_{b}=0.0.01$, $p_{c}=0.45$, and $p_{a}^{\prime }=0.25$, $p_{b}^{\prime }=0.05$, $p_{c}^{\prime }=0.70$. In this situation, we have that $H^{S}\left(A,D\right)=1.065<1.076=H^{S}\left(A,D^{\prime }\right)$, but $H^{T,2}\left(A,D\right)=0.506>0.445=H^{T,2}\left(A,D^{\prime }\right)$.*

On the other hand, if we consider the Renyi’s entropy measure when α goes to *∞*, we obtain

(20) $$H^{R,\infty }\left(A_{C},D\right)=-\log_{2}\left(\max\{p_{C1},p_{C2},\ldots ,p_{CK}\}\right),$$

and results similar to the above can be proved.

However, all Renyi’s entropy measures are correlated, therefore $H^{S}$, $H^{R,2}$, and $H^{R,\infty }$ are also correlated.

In view of the analysis above, the entropy of attribute $A_{C}$ is somehow caught by the random classifier X and the intuitive classifier V, in the sense that the higher the entropy, the lower the (expected) number of successes in the classification, and conversely. Therefore, the random classifier X and the intuitive classifier V can be used as benchmarks when evaluating other classifiers, taking into account the entropy of the consequent. Next we define an evaluation measure based on the analysis above.

**Definition** **1.** *Let Z be a classifier. Given a dataset D, and a consequent $A_{C}$, the performance of Z with respect to the random classifier X is given by*

(21) $$\gamma^{X}\left(Z\left(D\right)\right)=\left\{\begin{array}{cc} \frac{\mu (Z,D)-\mu (X,D)}{1-\mu (X,D)} & \mathrm{if}\mu (Z,D)-\mu (X,D)\geq 0\\ \; & \\ \frac{\mu (Z,D)-\mu (X,D)}{\mu (X,D)} & \mathrm{if}\mu (Z,D)-\mu (X,D)<0 \end{array}\right.,$$

*where $\mu \left(X,D\right)=\frac{E(\sum_{i=1}^{M}X\left(i\right))}{M}$, such that M is the total number of predictions, and μ(Z,D) is the ratio of correct classifications using classifier (Z).*

Note that the first case of the definition of the performance measure $\gamma^{X}$ coincides with the Scott’s π. If we use the intuitive classifier V instead of X as benchmark classifier, we obtain the performance measure $\gamma^{V}$. The evaluation measure $\gamma^{X}$ (resp. $\gamma^{V}$) runs between −1 and 1, where −1 is the worst case, and is achieved when the classifier does not predict correctly any instance; 0 means that performance is as the random classifier X (resp. $\gamma^{V}$); and 1 is the best case, and is achieved when the classifier correctly classifies all instances. The intermediate values measure in which proportion the classifier performs better (positive values) or worse (negative values) than the random classifier (resp. V).

On the other hand, we can interpret the performance measure $\gamma^{X}$ (resp. $\gamma^{V}$) in terms of proportional reduction of classification error with respect to the random classifier (resp. V). Indeed, if we predict *M* instances, we can write Equation (21) as follows:

(22) $$\gamma^{X}\left(Z\left(D\right)\right)=\left\{\begin{array}{cc} \frac{M\mu (Z,D)-M\mu (X,D)}{M-M\mu (X,D)} & \mathrm{if}M\mu (Z,D)-M\mu (X,D)\geq 0\\ \; & \\ \frac{M\mu (Z,D)-M\mu (X,D)}{M\mu (X,D)} & \mathrm{if}M\mu (Z,D)-M\mu (X,D)<0 \end{array}\right..$$

Now, we can write Equation (22) in the following way:

(23) $$\gamma^{X}\left(Z\left(D\right)\right)=\left\{\begin{array}{cc} \frac{\left(M-M\mu (X,D))-(M-M\mu (Z,D)\right)}{M-M\mu (X,D)} & \mathrm{if}M\mu (Z,D)-M\mu (X,D)\geq 0\\ \; & \\ \frac{M\mu (Z,D)-M\mu (X,D)}{M\mu (X,D)} & \mathrm{if}M\mu (Z,D)-M\mu (X,D)<0 \end{array}\right..$$

Finally, Equation (23) can be interpreted as follows:

(24) $$\gamma^{X}\left(Z\left(D\right)\right)=\left\{\begin{array}{cc} \frac{\mathrm{Expected}\;\#\;\mathrm{errors}\;\mathrm{by}\;\mathrm{using}\;X-\;\#\;\mathrm{errors}\;\mathrm{by}\;\mathrm{using}\;Z}{\mathrm{Expected}\;\#\;\mathrm{errors}\;\mathrm{by}\;\mathrm{using}\;X}, & \mathrm{if}M\mu (Z,D)-M\mu (X,D)\geq 0\\ \; & \\ \frac{\#\;\mathrm{successes}\;\mathrm{by}\;\mathrm{using}\;Z-\mathrm{Expected}\#\;\mathrm{successes}\;\mathrm{by}\;\mathrm{using}\;X}{\mathrm{Expected}\#\;\mathrm{successes}\;\mathrm{by}\;\mathrm{using}\;X} & \mathrm{if}M\mu (Z,D)-M\mu (X,D)<0 \end{array}\right..$$

Thus, the first case of $\gamma^{X}$ measures the proportional reduction of classification error when we use classifier Z with respect to using the random classifier X. The second case of $\gamma^{X}$ measures the proportional reduction of classification success when we use classifier Z with respect to using the random classifier X. The same can be said when using the intutitive classifier V as benchmark.

Therefore, $\gamma^{X}$ gives us information about how much a classifier Z improves or worsens the classification with respect to a classifier that decides the class randomly taking into account the frequency distribution of the classes. Furthermore, $\gamma^{X}$ gives us information about how much a classifier Z improves or worsens the classification with respect to a classifier that simply predicts the most likely class according to the frequency distribution of the classes. Since the previous two classifiers only use information related to the classes, these two measures provide information on whether it is relevant to use more sophisticated classifiers that incorporate information from other attributes.

On the other hand, the measure $\gamma^{X}$ and $\gamma^{V}$ incorporate in a way the information on the entropy of the consequent to the evaluation of a classifier, but do not take into account the rest of the attributes (the antecedents). Nevertheless, a similar analysis can be carried out by considering all possible different strings of attributes, obtaining analogous results. On the other hand, the intuitive classification method described in Section 2.2 can be another way of taking into account all the attributes and the entropy of the dataset, since its definition is based on the repetition of instances which is related to the entropy of the dataset. In particular, it is related to the conditional entropy of the attribute $A_{C}$ given the remaining variables in the dataset. Thus, another measure of evaluation of the classifiers related to entropy could be to use this intuitive classification method as a benchmark, its definition being analogous to those previously given. Below we formally outline the definition of this measure.

**Definition** **2.** *Let Z be a classifier. Given a dataset D, and a consequent $A_{C}$, the performance of Z with respect to the intuitive classifier I is given by*

(25) $$\Gamma \left(Z\left(D\right)\right)=\left\{\begin{array}{cc} \frac{\mu (Z,D)-\mu (I,D)}{1-\mu (I,D)} & \mathrm{if}\mu (Z,D)-\mu (I,D)\geq 0\\ \; & \\ \frac{\mu (Z,D)-\mu (I,D)}{\mu (I,D)} & \mathrm{if}\mu (Z,D)-\mu (I,D)<0 \end{array}\right.,$$

*where μ(I,D) is the ratio of correct classifications using classifier (I), and μ(Z,D) is the ratio of correct classifications using classifier (Z).*

The interpretation of Γ is completely analogous to that of γ above, only changing the random classifier X and the intuitive classifier V for the intuitive classifier I. However, it gives some extra information about classifiers, in the sense that since it uses all information in the dataset, it provides information on how much relevant is to use more sophisticated classifiers.

## 3. Computer-Based Experiments: Design and Results

In this section, we illustrate how the evaluation measures introduced in Section 2 work. For that end, we design an experiment in which we consider five scenarios of entropy for a binary attribute (the consequent), and for each of those scenarios we study 31 combinations of explanatory attributes (the antecedents). Thus, we can give a better idea about how these evaluation measures work and how they measure the performance of classifiers in different entropy situations. We then go further and carry out an extensive comparison for four classifiers by using 11 different datasets whose results are concisely presented.

### 3.1. Datasets and Scenarios

We start from the hypothesis of working in a classification context where the target to be predicted is discrete and more specifically binary, but another multi-class target variable could be considered. A well-known dataset from UCI Machine Learning Repository [87] named “thyroid0387.data” [88] has been chosen for the most intensive experiment.

This dataset has been widely used in the literature in problems related to the field of classification. Since it is only used in this paper as an example and we are not interested in the clinical topic itself that the data collect, in order to facilitate the experiment of this study and make it exhaustive, that dataset has been minimally preprocessed as follows:

- Headers have been added and renamed.
- The numeric attributes have been removed and we have left only those which are nominal.
- The class variable has been recoded in positive and negative cases (the original sample has several types of positive instances).

Finally, the dataset used to perform the experiment has the following features:

- Number of rows: 9173
- Number of attributes/columns: 23 (all nominal)
  **–**  22 explanatory variables (antecedents)
  **–**  1 target variable (consequent)
    * 2401 positive cases
    * 6772 negative cases

The target variable used to classify which corresponds to a clinical diagnosis, is unbalanced, as it has a positive value in 2401 tuples and a negative value in 6772. From these data we will consider five types of possible scenarios with different ratios between positive and negative values (see Table 1).

The remaining 10 datasets used in the most extensive experiment are also from UCI Machine Learning Repository [87]. The following modifications have been made, common to all of them.

1. In all the datasets that did not have a row with the header, it has been added, taking into account the specifications of the “Attribute Information” section of each of these UCI repository datasets.
2. The configuration in Weka to discretize has been with the parameter “bins” = 5 (to obtain 5 groups) and the parameter “UseEqualFrecuency” = true (so that the groups of data obtained were equitable).
3. When discretizing in Weka (filter→unsupervised→discretized) the results obtained were numerical intervals, so they were later renamed.

In particular, apart from the dataset already mentioned, we have used the following datasets:

- “Healthy_Older_People.data” [89,90];
- “Avila.data” [91,92];
- “Adult.data” [93];
- “nursery.data” [94];
- “Bank marketing” [95,96];
- “HTRU2.data” [97,98,99];
- “connect-4.data” [100];
- “tic-tac-toe.data” [101];
- “Credit approval.data” [102];
- “Mushroom.data” [103].

The main features of these datasets are summarized in Table 2.

In addition, some specific preprocessing of the data were carried out in the datasets “Adult.data” [93] and “Bank marketing” [95,96]. In “Adult.data”, the rows with missing values were removed, and three attributes were discarded (capital-gain, capital-loss, native-country); and in “Bank marketing”, the selected dataset was “bank-full.csv”, and 6 attributes were discarded (balance, day, duration, campaign, pdays, and previous).

### 3.2. Experimental Design

The experiment consists of determining the accuracy of an heuristic classifier, the already mentioned J48, in comparison with three benchmark classifiers: the random classifier and two intuitive classifiers. These three classifiers to certain extent contain information about the entropy present in the dataset as explained in the previous section. Therefore, we provide evaluation measures of that classifier taking into account the entropy of the system. In this sense, we try to evaluate how this classifier performs in terms of the improvement (or deterioration) obtained with respect to three classifiers that can be considered as benchmarks and that are based on the simple distribution of data from the dataset, and then on the entropy of the data.

On the other hand, we are also interested in observing the differences between the three evaluation measures of the classifiers introduced in the previous section, and what effect, considering more or less information from the dataset, this has when making classifications of instances. To do this, we consider the five scenarios described in Table 1, which have different level of Shannon’s entropy in the consequent. For each of these scenarios, we follow the process depicted in Figure 1.

First, starting from original sample of data and fixing the consequent variable (or target variable) $A_{C}$ to be studied, the five variables (attributes) more correlated with the target variable are selected. Then they are sorted $\left(A_{1},A_{2},A_{3},A_{4},A_{5}\right)$, that is, we determine which is more correlated with the consequent and which less, for which we use the *gain ratio attribute method* described in Section 2.1. In Table 3, we show the gain ratio scores observed for each of the five scenarios (S1,S2,S3,S4,S5) considered.

At this point, we would like to emphasize once again that it is not our purpose to analyze a particular problem, but only to use a dataset for analyzing the evaluation measures introduced in this paper and also show an analysis of heuristic classifiers when considering entropy characteristics of the dataset. For this reason, attributes $A_{1},\ldots ,A_{5}$ are not necessarily the same nor they are in the same order in the five scenarios. We simply call generically $A_{1}$ to the attribute best correlated with the target variable in each scenario, even if it is not the same variable in each of them. Accordingly, the other attributes occupy second to fifth positions in the correlation ranking with the consecutive attribute in each scenario, always according to the gain ratio attribute evaluation. In each of the scenarios, these five attributes will be used as predictor or explanatory variables (antecedents) to generate the classification models. It is not an objective of this work to delve into the different methods of features (attributes) subset selection, but we simply use one of them, always the same (gain ratio attribute), in order to work only with those attributes that in each case are really significant. Reducing the size of the problem from 22 to 5 explanatory variables will allow a comprehensive experiment with which to illustrate and analyze the two introduced evaluation measures, and to show a way to analyze the performance of an heuristic classifier when we consider different degrees of entropy in the dataset. In order to select the five best attributes, we use the software Weka [75,82,83], in particular, its *Select attributes* function, with *GainRatioAttributeEval* as the attribute evaluator, *ranker* as the search method, and *cross-validation* as attribution selection mode. Note that Weka gives two measures of the relevance of the (antecedent) attributes. The average merit and its standard deviation, and the average rank and its standard deviation. The first refers to the mean of the correlations measured with GainRatioAttributeEval in 10 cycles (although with 5 cycles would have been sufficient, since only the first 5 attributes are wanted) of validation fold. The average rank refers to the average order in which each attribute remained in each of the ten cycles. See [75,82] for details about Weka.

Once the five best attributed are chosen, the next step is to establish the 31 possible combinations of the set of predictor variables. These 31 combinations will be the background to consider in a set of classification rules or in a decision tree. That is, 31 classification studies will be carried out to predict the consequent attribute $A_{C}$ based on each of these combinations of explanatory variables (see Table 4).

For each of these attribute combinations we generate 100 subsamples to avoid possible biases in the selection of records.

Third, for each of the scenarios described (Table 1), for each of the 31 combinations of antecedent attributes (Table 4), and for each of the 100 random subsamples, classification models are generated, both with the two intuitive classifiers and with the heuristic method J48. Thus, we have carried out 15,500 heuristic classification models with the J48 method as well as with our own implementation of the intuitive classifier I.

Finally, for both classifiers we calculate their accuracies, from their corresponding confusion matrices by using cross-validation. Therefore, to calculate the success ratio μ(X,D) of the random classifier X, we directly use the theoretical result given by Equation (7), and the same for the intuitive classifier V using Equation (12), while to calculate the success ratio μ(I,D) of the intuitive classifier I, we use the confusion matrix obtained by cross-validation. Likewise, the success ratio μ(Z,D) of the heuristic classifier, in our case J48, is also calculated by the confusion matrix obtained by cross-validation. From these results, the evaluation measures introduced in Section 2.4 can already be calculated.

Therefore, we have an experimental design with two factors (entropy scenarios and attribute combinations) with 100 replications for each cross combination of factors. This allows us to analyze in depth how an heuristic classifier performs when we consider both the entropy of the consequent variable and the number of attributes used as antecedents.

Therefore, the experiment illustrates both how the evaluation measures work and how to analyze the effects of entropy and the number of selected attributes to predict the consequent variable in the performance of an heuristic classifier.

### 3.3. Results

After performing all the classification models described in the previous section for each of the five scenarios, each model is subjected to a cross-validation test, and confusion matrices are determined. With this information we can calculate some performance measures for the heuristic classifier J48. The simplest performance measure is accuracy, which measures the success rate in the prediction. Table 5 shows the accuracy of J48 and the intuitive classifier I for each of the five scenarios considered.

In Table 5, we observe that, for this dataset, the performance of J48 is on average slightly better than the performance of the intuitive classifier I, but the mean absolute errors for J48 are worse than the mean absolute errors of the intuitive classifier I except for S5. However, this comparison could be analyzed in more detail considering other aspects such as the number of times that one method beats the other or the entropy. Likewise, the improvements with respect to the intuitive classifier V are not too great, which would mean that either the model is not very good, or that in this specific case the use of information from other attributes and/or classifiers more sophisticated do not provide noticeable improvements over the intuitive classifier V.

We now consider that a classifier beats another classifier each time that the first correctly classifies a number of items from the test set higher than the items correctly classified by second. When the reverse occurs, we will say that the second classifier beats the first. When the difference between the items well classified by both methods is 0, we will say that a draw has occurred. The number of times that J48 and the intuitive classifier I win for each scenario and each combination of the best five attributes are shown in Table A1, Table A2, Table A3, Table A4 and Table A5 in Appendix A. Table 6 summarizes the percentage of times each method wins for each scenario.

In Table 6, we observe that J48 classifies better than the intuitive method I in 47.48% of the instances, while the intuitive method I classifies better than J48 in 24.63% of the instances. J48 classifies particularly better in scenarios S5 and S3, while the intuitive method I classifies better in scenarios S2 and S4. Moreover, J48 clearly beats the intuitive classifier V in all scenarios except in S1, while the intuitive method I classifies better than the intuitive classifier V in scenarios S2, S4 and S5. Therefore, in absolute terms we can say that J48 performs reasonably well with respect to the dataset used. However, in addition to knowing whether one method classifies better than another, it is even more relevant to know how much better it classifies in relative terms as mentioned above. In this sense, having a benchmark is important to assess how much improvement there is when compared to it. In Table A1, Table A2, Table A3, Table A4 and Table A5 in Appendix A, we can find the evaluation measures introduced in Section 2.4 applied to the average of the results obtained for the 100 subsamples for each combination of the best attributes when J48 and the intuitive classifier are used. Table 7 summarizes these measures for each of the five scenarios considered.

First note that in this case the measure $\gamma^{X}$ coincides in all scenarios with the Scott’s π. On the other hand, beyond that which was analyzed when we evaluate which method best classifies simply in terms of the number of successes, in Table 7 we observe that the performance of J48 and the intuitive classifier I are very similar when compared with the random classifier X and the intuitive classifier V for each of the scenarios (columns corresponding to evaluation measures $\gamma^{X}$ and $\gamma^{V}$). This is clearly reflected in the evaluation measure Γ of J48, which is the result of comparison with the intuitive method I (see Definition 2). We also observe that, for the dataset used in the experiment, the performance of the classifiers improves with the decrease in the entropy of the consequent, i.e., the lower the entropy, the higher the performance of both classifiers with respect to the random classifier X.

Moreover, if we look, for example, at scenario S3, $\gamma^{V}\left(J48\right)$ tells us that J48 improves the performance of the intuitive classifier V, which only uses the information provided by the frequency distribution of the target attribute, by as much as 5% using the information provided by attributes other than the target attribute. Therefore, this percentage can be interpreted as the exploitation that J48 makes of this additional information. If we now look at Γ(J48), then we see that this improvement reaches almost 8.5% with respect to the intuitive classifier I. This percentage can be interpreted as the better exploitation that J48 makes of the information than the intuitive classifier I. At this point, one could already assess, taking into account the practical implications of better performance, whether the use of a more sophisticated classifier than the two intuitive classifiers is worth it.

Therefore, comparison with a benchmark is important because performance measures often do not reflect what is actually gained with respect to a simple, even random, way of classifying. Therefore, the use of measures based on simple benchmark classifiers that somehow capture the entropy of the dataset seems appropriate and provides relevant information on the performance of the classifiers. In particular, the use of both intuitive classifiers as benchmark seems reasonable, because although as classifiers they have been discarded in favor of other classifiers that use more modern and elaborate technologies, they are still easy enough to understand and intuitive as to at least consider them as benchmark classifiers when measuring the performance of classifiers, as the random classifier is commonly used in machine learning.

### 3.4. Extensive Experiment

In this subsection we present the results of an extensive experiment in which we consider four heuristic classifiers besides the intuitive classifier I, and 11 datasets. In particular, we consider four classification algorithms implemented in Weka [75,81,82,83], J48, Naïve Bayes, SMO, and Random Forest, which have been briefly described in Section 2.3; and 11 datasets from UCI Machine Learning Repository [87] which have been described in Section 3.1.

The purpose of this extensive analysis is to check whether the results obtained in the previous experiment are repeated for other classifiers and other datasets. The first step in all cases is to select the 5 most relevant attributes by using the feature selection method described in Section 2.1. The results are shown in Table 8.

Then the five classifiers are applied with the selection of attributes in Table 8. We calculate their accuracies, from their corresponding confusion matrices by using cross-validation. The resulting accuracies for each classifier and dataset are shown in Table 9.

In Table 10 and Table 11, we present the results obtained when $\gamma^{X}$ and $\gamma^{X}$ are used as evaluation performance measure.

As we mentioned before, we know that the $\gamma^{X}$ measure is close related to the κ and π measures. In Table 10 and Table 11, we observe that a higher entropy in the consequent attribute does not mean a worse performance of the classifiers [70]. This is not surprising since all classifiers use not only the frequency distribution information of the consequent attribute, but also the information provided about it by the remaining attributes in the dataset. Therefore, it seems appropriate to use the entropy of the entire dataset as a reference when assessing the performance of the classifiers. This entropy is somehow captured by the intuitive classifier I as explained earlier. In Table 12, we present the results obtained when Γ is used as evaluation performance measure.

The intuitive classifier I will have better accuracy the lower the conditional entropy of the target attribute given the entire dataset (or the subset of selected attributes if a selection feature is previously carried out), therefore, it will be more difficult for a classifier to significantly improve the classification results of this intuitive classifier. On the other hand, it is necessary to emphasize that the selection of the best subset of attributes has been relevant throughout the classification process, since the method used is based on the reduction of entropy. In this sense, Γ would measure how much a classifier contributes to the complete classification procedure with respect to what is contributed by the attribute selection process. Therefore, Γ offers different information than other performance measures of the classifiers, which we consider to be interesting. The aim, therefore, is not to substitute for any known performance measure, but to provide a measure of a different aspect of the performance of a classifier.

Finally, in Table 11 and Table 12, we observe that performance measures $\gamma^{V}$ and Γ provide complementary information about classifiers. In Table 11, we can observe how each classifier takes advantage of the information provided by the attributes in the dataset to better classify the target attribute, while in Table 12 we can observe how much better than the intuitive classifier I are classifiers capable of using the information in the dataset to correctly predict the classes of the target attribute.

## 4. Discussion and Conclusions

In the experiment we have shown that both feature selection and the entropy of the consequent attribute may be relevant to the performance result of an algorithm of classification. Therefore, it would appear to be of interest to consider the diversity of the response variable or the dataset when evaluating a classifier. In addition, the effect of entropy is observed, in the sense that the lower the entropy, the higher the success rate in the classifications, which seems intuitively reasonable. On the other hand, we observe in the experiment that choosing a greater number of features does not always provide a better performance of the classification algorithm, so this kind of analysis is relevant when selecting an adequate number of features, above all when the feature selection algorithm has not used the classifier algorithm for optimal selection. A rigorous analysis of the latter can be found in [104].

The performance measures of classifiers which only use the results of the classification algorithm itself, such as the ratio of successes (accuracy), do not really provide information on how it is really capable of classifying correctly with respect to unsophisticated methods. For this reason, the use of relative measures when compared with simple benchmark classifiers is important, because they give us information about the relationship between the gain in the correct classification of instances and the effort made in the design of new classifiers with respect to the use of simple and intuitive classifiers, i.e., we can better assess the real improvement provided by the classification algorithm. Moreover, if the benchmark classifier incorporates some type of additional information, such as different aspects of the entropy of all the dataset or the consequent attribute, the information provided by the performance measure will be even more relevant.

In this paper, three simple classifiers have been used, the random classifier X, the intuitive classifier V, and the intuitive classifier I. The first two simply use the distribution of the consequent attribute to classify and we have shown that they are closely related to the entropy of that attribute, while the third uses the entire distribution of the whole data set to classify and its performance is close to the conditional entropy of the consequent attribute given the remaining attributes (or a subset of attributes if feature selection is previously applied) in the dataset. These three classifiers have been used as references to introduce three measures of the performance of classifiers. These measure how much a classifier improves (or worsens) over these simple classifiers that are related to certain aspects of the entropy of the consequent attribute within the dataset. Therefore, they are measures that reflect on the performance of the heuristic classifiers, taking into account entropy in some way, and this is important, because the greater the entropy, the greater the difficulty to classify correctly, as has been seen in the experiment, which gives a better idea of the true performance of a classifier. Likewise, the three performance measures of classifiers can be interpreted in terms of proportional reduction of the classification error, which makes these measures easily understandable. In particular, $\gamma^{X}$ is closely related to the well-known κ and π measures, and provides information on how much a classifier improves the classification results relative to a random classifier that it only takes into account the information contained in the frequency distribution of the target attribute classes. $\gamma^{V}$ gives information on how a classifier is capable to use the information contained in the whole dataset (or a subset of the dataset) to improve the classification results relative to a classifier that it only uses the information of the frequency distribution of the target attribute classes and always predicts the most likely class. Last, Γ provides information on how much a classifier improves the classification results when using a more elaborate technology of managing data than the intuitive classifier I which simply predicts the most likely class given a particular profile of attributes in the dataset.

To conclude, although the two intuitive classifiers used in this paper were already discarded in favor of more modern and sophisticated classifiers, we believe that they are still useful as benchmark classifiers, as the random classifier is commonly used in machine learning, and then to design performance measures based on them which we have shown throughout this work that provide relevant information about the performance of classifiers different from other performance measures.

## Figures and Tables

**Table 1:** The five data scenarios.

Scenario	Positive	Negative	Total	Ratio Positive/Negative	Consequent’s Entropy
S1	2400	800	3200	3:1	0.811
S2	2400	1200	3600	2:1	0.918
S3	2400	2400	4800	1:1	1.000
S4	2000	4000	6000	1:2	0.918
S5	2000	6000	8000	1:3	0.811

**Table 2:** Main features of the datasets. **# rows** means the number of rows of the dataset; **# attributes** means the number of attributes including the consequent, and below the type of variables; **# classes** is the number of classes of the consequent; and **Distribution of the classes** is the number of cases of each class in the dataset.

Dataset	# Rows	# Attributes	# Classes	Distribution of the Classes
Thyroid	9173	23 2 categorical 21 binary	2	2401, 6772
Healthy	75,128	10 8 real 1 binary 1 categorical	4	16406, 4911, 51520, 2291
Avila	20,867	11 10 real 1 categorical	12	8572, 10, 206, 705, 2190, 3923, 893, 1039, 1663, 89, 1044, 533
Adult	32,561	12 3 real 1 integer 6 categorical 2 binary	2	7841, 24720
Nursery	12,960	9 8 categorical 1 binary	5	4320, 4266, 24044, 328
Bank	45,211	11 1 real 1 integer 5 categorical 4 binary	2	39922, 5289
HTRU2	17,898	9 8 real 1 binary	2	16259, 1639
Connect-4	67,557	43 43 categorical	3	6449, 16635, 44473
Tic-tac-toe	958	10 9 categorical 1 binary	2	332, 626
Credit	690	10 5 categorical 5 binary	2	383, 307
Mushroom	8124	23 17 categorical 6 binary	2	4208, 3916

**Table 3:** Results of gain ratio attribute evaluation in the five scenarios.

Attributes	S1	S2	S3	S4	S5
$A_{1}$	0.036	0.050	0.083	0.122	0.102
$A_{2}$	0.037	0.037	0.082	0.076	0.134
$A_{3}$	0.033	0.034	0.028	0.020	0.016
$A_{4}$	0.034	0.032	0.028	0.015	0.013
$A_{5}$	0.029	0.022	0.026	0.013	0.010

**Table 4:** The 31 combinations of the best five attributes $A_{1}$, $A_{2}$, $A_{3}$, $A_{4}$, and $A_{5}$ for predicting consequent $A_{C}$.

Comb.	Antecedents	Comb.	Antecedents	Comb.	Antecedents
#1	$A_{5}$	#12	$A_{2},A_{3}$	#23	$A_{1},A_{3},A_{4},A_{5}$
#2	$A_{4}$	#13	$A_{2},A_{3},A_{5}$	#24	$A_{1},A_{2}$
#3	$A_{4},A_{5}$	#14	$A_{2},A_{3},A_{4}$	#25	$A_{1},A_{2},A_{5}$
#4	$A_{3}$	#15	$A_{2},A_{3},A_{4},A_{5}$	#26	$A_{1},A_{2},A_{4}$
#5	$A_{3},A_{5}$	#16	$A_{1}$	#27	$A_{1},A_{2},A_{4},A_{5}$
#6	$A_{3},A_{4}$	#17	$A_{1},A_{5}$	#28	$A_{1},A_{2},A_{3}$
#7	$A_{3},A_{4},A_{5}$	#18	$A_{1},A_{4}$	#29	$A_{1},A_{2},A_{3},A_{5}$
#8	$A_{2}$	#19	$A_{1},A_{4},A_{5}$	#30	$A_{1},A_{2},A_{3},A_{4}$
#9	$A_{2},A_{5}$	#20	$A_{1},A_{3}$	#31	$A_{1},A_{2},A_{3},A_{4},A_{5}$
#10	$A_{2},A_{4}$	#21	$A_{1},A_{3},A_{5}$		
#11	$A_{2},A_{4},A_{5}$	#22	$A_{1},A_{3},A_{4}$		

**Table 5:** Accuracy measures for the random classifier X, the intuitive classifier V, J48 and the intuitive classifier I when using combination of attributes A31 for each scenario. The accuracy and the mean absolute error are calculated as the average accuracy and the average mean absolute error of the 100 subsamples. Results are presented as *accuracy* ± *mean absolute error*.

Scenario	E(acc(X(D)))	acc(V(D))	acc(J48(D))	acc(I(D))
S1	0.6250	0.7500 ± 0.2500	0.7489 ± 0.3739	0.7481 ± 0.2519
S2	0.5556	0.6667 ± 0.3333	0.6724 ± 0.4358	0.6729 ± 0.3271
S3	0.5000	0.5000 ± 0.5000	0.5241 ± 0.4856	0.4835 ± 0.5165
S4	0.5556	0.6667 ± 0.3333	0.6751 ± 0.4366	0.6766 ± 0.3234
S5	0.6250	0.7465 ± 0.2535	0.7543 ± 0.3734	0.7537 ± 0.2487

**Table 6:** Percentage of times each method wins in each of the 3100 instances considered (100 subsamples for each of the 31 combinations of the best five attributes) for each scenario given in Table 1.

Scenario	J48 wins	V wins	J48 wins	I wins	I wins	V wins
S1	10.42	41.71	46.03	23.94	18.39	58.13
S2	67.65	15.65	24.48	37.39	74.90	13.29
S3	97.55	0.16	73.48	4.45	32.52	66.90
S4	78.13	0.26	16.52	42.19	84.52	0.00
S5	98.03	1.23	76.90	15.16	97.29	2.00
Average %	70.36	11.80	47.48	24.63	61.52	28.06

**Table 7:** Intervals of values of evaluation measure $\gamma^{X}$ for J48 and the intuitive method I, and intervals of values of evaluation measure Γ for J48 for each scenario.

Scenario	$\gamma^{X}\left(J48\right)$	$\gamma^{X}\left(I\right)$	$\gamma^{V}\left(J48\right)$	$\gamma^{V}\left(I\right)$	Γ(J48)
S1	0.3303–0.3333	0.3282–0.3333	−0.0015–0.0000	−0.0025–0.0000	0.0000–0.0032
S2	0.2499–0.2636	0.2498–0.2650	−0.0001–0.0182	−0.0001–0.0200	−0.0009–0.0001
S3	0.0004–0.0492	−0.0903–0.0419	0.0004–0.0492	−0.0903–0.0419	0.0049–0.0844
S4	0.2500–0.2693	0.2500–0.2729	0.0000–0.0257	0.0000–0.0306	−0.0026–0.0004
S5	0.3134–0.3635	0.3125–0.3627	−0.0087–0.0524	−0.0091–0.0512	0.0010–0.0027

**Table 8:** The five most relevant attributes of each dataset according to Gain Ratio Attribute Evaluation (see Section 2.1).

#	Dataset	1st	2nd	3rd	4th	5th
1	Thyroid	hypopit.	pregnant	psych	goitre	referral_
						source
2	Healthy	C4	C3	C6	C5	C7
3	Avila	F5	F1	F9	F3	F7
4	Adult	Mar.Sta.	Relat.	Sex	Age	Educ
5	Nursery	F2	F1	F7	F5	F4
6	Bank	poutcome	contact	housing	month	loan
7	HTRU2	A3	A1	A4	A6	A5
8	Connect-4	g6	d3	f6	d2	b6
9	Tic-tac-toe	m-m-s	b-l-s	t-l-s	t-r-s	b-r-s
10	Credit	A9	A10	A4	A5	A6
11	Mushroom	odor	gill-size	stalk-surface-	spore-print-	ring-type
				above-ring	color	

**Table 9:** Accuracies and mean absolute errors for the five classifiers and the 11 datasets. Results are presented as *accuracy* ± *mean absolute error*.

#	Dataset	I	J48	SMO	Naïve Bayes	Random Forest
1	Thyroid	0.743 ± 0.257	0.744 ± 0.381	0.743 ± 0.257	0.741 ± 0.373	0.743 ± 0.374
2	Healthy	0.953 ± 0.024	0.963 ± 0.030	0.949 ± 0.255	0.935 ± 0.042	0.963 ± 0.028
3	Avila	0.653 ± 0.058	0.666 ± 0.074	0.600 ± 0.141	0.610 ± 0.087	0.657 ± 0.069
4	Adult	0.825 ± 0.175	0.824 ± 0.250	0.818 ± 0.182	0.763 ± 0.240	0.824 ± 0.236
5	Nursery	0.508 ± 0.197	0.548 ± 0.224	0.508 ± 0.265	0.531 ± 0.233	0.508 ± 0.224
6	Bank	0.885 ± 0.115	0.894 ± 0.186	0.893 ± 0.107	0.890 ± 0.175	0.893 ± 0.167
7	HTRU2	0.971 ± 0.029	0.971 ± 0.049	0.969 ± 0.031	0.969 ± 0.050	0.971 ± 0.048
8	Connect-4	0.658 ± 0.228	0.665 ± 0.313	0.665 ± 0.318	0.663 ± 0.318	0.665 ± 0.311
9	Tic-tac-toe	0.801 ± 0.193	0.794 ± 0.258	0.753 ± 0.247	0.753 ± 0.374	0.840 ± 0.209
10	Credit	0.859 ± 0.141	0.862 ± 0.220	0.858 ± 0.142	0.861 ± 0.193	0.848 ± 0.199
11	Mushroom	1.000 ± 0.000	1.000 ± 0.000	0.999 ± 0.001	0.999 ± 0.020	1.000 ± 0.000

**Table 10:** Evaluation measure $\gamma^{X}$ for the five classifiers and the 11 datasets, and [0,1]-normalized Shannon entropy of the consequent attribute for each dataset.

#	Dataset	Entropy	$\gamma^{X}\left(I\right)$	$\gamma^{X}\left(J48\right)$	$\gamma^{X}\left(\mathrm{SMO}\right)$	$\gamma^{X}\left(\mathrm{NB}\right)$	$\gamma^{X}\left(\mathrm{RF}\right)$
1	Thyroid	0.829	0.335	0.338	0.335	0.330	0.335
2	Healthy	0.632	0.901	0.922	0.893	0.864	0.922
3	Avila	0.737	0.549	0.566	0.480	0.493	0.554
4	Adult	0.796	0.521	0.519	0.502	0.352	0.519
5	Nursery	0.739	0.279	0.338	0.279	0.313	0.279
6	Bank	0.521	0.443	0.487	0.482	0.468	0.482
7	HTRU2	0.442	0.826	0.826	0.814	0.814	0.826
8	Connect-4	0.769	0.312	0.326	0.326	0.322	0.326
9	Tic-tac-toe	0.931	0.561	0.545	0.455	0.455	0.647
10	Credit	0.991	0.715	0.721	0.713	0.719	0.692
11	Mushroom	0.999	1.000	1.000	0.998	0.998	1.000

**Table 11:** Evaluation measure $\gamma^{V}$ for the five classifiers and the 11 datasets, and the accuracy of the intuitive classifier X.

#	Dataset	acc(V)	$\gamma^{V}\left(I\right)$	$\gamma^{V}\left(J48\right)$	$\gamma^{V}\left(\mathrm{SMO}\right)$	$\gamma^{V}\left(\mathrm{NB}\right)$	$\gamma^{V}\left(\mathrm{RF}\right)$
1	Thyroid	0.738	0.018	0.022	0.018	0.011	0.018
2	Healthy	0.686	0.850	0.882	0.838	0.793	0.882
3	Avila	0.411	0.411	0.433	0.321	0.338	0.418
4	Adult	0.759	0.273	0.269	0.244	0.016	0.269
5	Nursery	0.333	0.262	0.322	0.262	0.297	0.262
6	Bank	0.883	0.017	0.094	0.085	0.060	0.085
7	HTRU2	0.908	0.683	0.683	0.661	0.661	0.683
8	Connect-4	0.658	0.000	0.020	0.020	0.014	0.020
9	Tic-tac-toe	0.653	0.426	0.406	0.287	0.287	0.538
10	Credit	0.555	0.683	0.690	0.681	0.688	0.658
11	Mushroom	0.518	1.000	1.000	0.998	0.998	1.000

**Table 12:** Evaluation measure Γ for the four heuristic classifiers and the 11 datasets.

#	Dataset	Γ(J48)	Γ(SMO)	Γ(NB)	Γ(RF)
1	Thyroid	0.004	0.000	−0.003	0.000
2	Healthy	0.213	−0.004	−0.019	0.213
3	Avila	0.037	−0.081	−0.066	0.012
4	Adult	−0.001	−0.008	−0.075	−0.001
5	Nursery	0.081	0.000	0.047	0.000
6	Bank	0.078	0.070	0.043	0.070
7	HTRU2	0.000	−0.002	−0.002	0.000
8	Connect-4	0.020	0.020	0.015	0.020
9	Tic-tac-toe	−0.009	−0.060	−0.060	0.196
10	Credit	0.021	−0.001	0.014	−0.013
11	Mushroom	0.000	−0.001	−0.001	0.000

## Data Availability

The datasets used in the experiments can be found at UCI Machine Learning Repository: https://archive.ics.uci.edu/ml/index.php (accessed on 23 April 2021).

## Appendix A. Tables

**Table A1:** Scenario S1, 3.200 rows, 3:1 ratio of positive/negative values for target variable, 100 subsamples per combination, and the gain ratio attribute evaluations of the five best variables are 0.036, 0.037, 0.033, 0.034, and 0.029 (from most to least relevant).

Comb.	Antecedents	J48 wins	I wins	$\gamma^{X}\left(J48\right)$	Γ(J48)	$\gamma^{X}\left(I\right)$	$\gamma^{V}\left(J48\right)$	$\gamma^{V}\left(I\right)$
#1	5	45	10	0.3328	0.0019	0.3316	−0.0003	−0.0009
#2	4	39	33	0.3326	0.0003	0.3324	−0.0004	−0.0005
#3	45	56	28	0.3320	0.0022	0.3306	−0.0007	−0.0014
#4	3	39	16	0.3318	0.0024	0.3301	−0.0008	−0.0016
#5	35	55	33	0.3314	0.0031	0.3294	−0.0010	−0.0020
#6	34	57	30	0.3311	0.0029	0.3291	−0.0011	−0.0021
#7	345	56	34	0.3305	0.0032	0.3284	−0.0014	−0.0025
#8	2	0	0	0.3333	0.0000	0.3333	0.0000	0.0000
#9	25	45	10	0.3328	0.0019	0.3316	−0.0003	−0.0009
#10	24	44	28	0.3326	0.0005	0.3322	−0.0004	−0.0006
#11	245	61	26	0.3321	0.0025	0.3304	−0.0006	−0.0015
#12	23	47	24	0.3316	0.0021	0.3302	−0.0009	−0.0016
#13	235	55	36	0.3312	0.0027	0.3294	−0.0011	−0.0020
#14	231	56	32	0.3307	0.0024	0.3291	−0.0013	−0.0021
#15	2345	58	33	0.3303	0.0030	0.3282	−0.0015	−0.0025
#16	1	0	0	0.3333	0.0000	0.3333	0.0000	0.0000
#17	15	45	10	0.3328	0.0019	0.3316	−0.0002	−0.0009
#18	14	40	32	0.3326	0.0004	0.3324	−0.0004	−0.0005
#19	145	57	27	0.3321	0.0023	0.3306	−0.0006	−0.0014
#20	13	39	16	0.3318	0.0024	0.3301	−0.0008	−0.0016
#21	135	53	33	0.3312	0.0028	0.3294	−0.0011	−0.0020
#22	134	55	31	0.3310	0.0028	0.3291	−0.0012	−0.0021
#23	1345	58	33	0.3305	0.0032	0.3284	−0.0014	−0.0025
#24	12	0	0	0.3333	0.0000	0.3333	0.0000	0.0000
#25	125	45	10	0.3328	0.0019	0.3316	−0.0003	−0.0009
#26	124	44	28	0.3327	0.0007	0.3322	−0.0003	−0.0006
#27	1245	62	25	0.3321	0.0025	0.3304	−0.0006	−0.0015
#28	123	47	24	0.3316	0.0022	0.3302	−0.0009	−0.0016
#29	1235	55	35	0.3311	0.0026	0.3294	−0.0011	−0.0020
#30	1234	57	31	0.3308	0.0026	0.3291	−0.0013	−0.0021
#31	12345	57	34	0.3303	0.0031	0.3282	−0.0015	−0.0025
	**Total**	1427	742					
	**%**	46.03	23.94					

**Table A2:** Scenario S2, 3.600 rows, 2:1 ratio of positive/negative values for target variable, 100 subsamples per combination, and the gain ratio attribute evaluations of the five best variables are 0.050, 0.037, 0.034, 0.032, and 0.022 (from most to least relevant).

Comb.	Antecedents	J48 wins	I wins	$\gamma^{X}\left(J48\right)$	Γ(J48)	$\gamma^{X}\left(I\right)$	$\gamma^{V}\left(J48\right)$	$\gamma^{V}\left(I\right)$
#1	5	38	35	0.2499	0.0000	0.2498	−0.0001	−0.0001
#2	4	7	34	0.2526	−0.0004	0.2532	0.0034	0.0043
#3	45	35	45	0.2525	−0.0003	0.2530	0.0034	0.0041
#4	3	1	12	0.2611	−0.0004	0.2617	0.0148	0.0156
#5	35	43	34	0.2604	−0.0002	0.2607	0.0139	0.0143
#6	34	6	41	0.2636	−0.0008	0.2649	0.0182	0.0198
#7	345	33	54	0.2625	−0.0008	0.2638	0.0167	0.0184
#8	2	1	0	0.2500	0.0000	0.2500	0.0000	0.0000
#9	25	38	35	0.2499	0.0000	0.2498	−0.0001	−0.0001
#10	24	31	34	0.2524	−0.0003	0.2529	0.0032	0.0038
#11	245	41	46	0.2523	−0.0003	0.2527	0.0030	0.0036
#12	23	9	46	0.2612	−0.0007	0.2623	0.0150	0.0163
#13	235	37	48	0.2605	−0.0005	0.2612	0.0140	0.0150
#14	231	26	59	0.2636	−0.0009	0.2650	0.0181	0.0200
#15	2345	34	57	0.2628	−0.0008	0.2640	0.0171	0.0187
#16	1	0	0	0.2500	0.0000	0.2500	0.0000	0.0000
#17	15	38	35	0.2499	0.0000	0.2498	−0.0001	−0.0001
#18	14	7	34	0.2525	−0.0004	0.2532	0.0034	0.0043
#19	145	33	47	0.2524	−0.0004	0.2530	0.0032	0.0041
#20	13	1	12	0.2612	−0.0004	0.2617	0.0149	0.0156
#21	135	43	34	0.2605	−0.0002	0.2607	0.0140	0.0143
#22	134	6	41	0.2636	−0.0008	0.2649	0.0182	0.0198
#23	1345	36	51	0.2628	−0.0007	0.2638	0.0170	0.0184
#24	12	0	0	0.2500	0.0000	0.2500	0.0000	0.0000
#25	125	38	35	0.2499	0.0001	0.2498	−0.0001	−0.0001
#26	124	31	34	0.2524	−0.0003	0.2529	0.0032	0.0038
#27	1245	41	46	0.2523	−0.0002	0.2527	0.0031	0.0036
#28	123	9	45	0.2612	−0.0007	0.2623	0.0150	0.0163
#29	1235	37	48	0.2605	−0.0005	0.2612	0.0140	0.0150
#30	1234	25	60	0.2636	−0.0009	0.2650	0.0182	0.0200
#31	12345	34	57	0.2628	−0.0008	0.2640	0.0170	0.0187
	**Total**	759	1159					
	**%**	24.48	37.39					

**Table A3:** Scenario S3, 4.800 rows, 1:1 ratio of positive/negative values for target variable, 100 subsamples per combination, and the gain ratio attribute evaluations of the five best variables are 0.083, 0.082, 0.028, 0.028, and 0.026 (from most to least relevant).

Comb.	Antecedents	J48 wins	I wins	$\gamma^{X}\left(J48\right)$	Γ(J48)	$\gamma^{X}\left(I\right)$	$\gamma^{V}\left(J48\right)$	$\gamma^{V}\left(I\right)$
#1	5	72	1	0.0365	0.0390	−0.0026	0.0365	−0.0026
#2	4	100	0	0.0076	0.0839	−0.0833	0.0076	−0.0833
#3	45	33	0	0.0448	0.0173	0.0279	0.0448	0.0279
#4	3	100	0	0.0067	0.0842	−0.0846	0.0067	−0.0846
#5	35	49	4	0.0417	0.0259	0.0161	0.0417	0.0161
#6	34	100	0	0.0140	0.0833	−0.0756	0.0140	−0.0756
#7	345	18	5	0.0492	0.0076	0.0419	0.0492	0.0419
#8	2	18	0	0.0323	0.0070	0.0255	0.0323	0.0255
#9	25	100	0	0.0343	0.0797	−0.0493	0.0343	−0.0493
#10	24	60	11	0.0324	0.0253	0.0074	0.0324	0.0074
#11	245	100	0	0.0436	0.0806	−0.0402	0.0436	−0.0402
#12	23	57	0	0.0323	0.0235	0.0090	0.0323	0.0090
#13	235	100	0	0.0399	0.0805	−0.0441	0.0399	−0.0441
#14	231	86	2	0.0324	0.0470	−0.0153	0.0324	−0.0153
#15	2345	99	0	0.0487	0.0781	−0.0319	0.0487	−0.0319
#16	1	100	0	0.0004	0.0832	−0.0903	0.0004	−0.0903
#17	15	77	0	0.0372	0.0433	−0.0064	0.0372	−0.0064
#18	14	100	0	0.0075	0.0838	−0.0832	0.0075	−0.0832
#19	145	37	0	0.0448	0.0190	0.0262	0.0448	0.0262
#20	13	100	0	0.0071	0.0844	−0.0845	0.0071	−0.0845
#21	135	50	4	0.0417	0.0274	0.0147	0.0417	0.0147
#22	134	100	0	0.0140	0.0832	−0.0755	0.0140	−0.0755
#23	1345	19	5	0.0492	0.0081	0.0414	0.0492	0.0414
#24	12	15	45	0.0325	0.0049	0.0277	0.0325	0.0277
#25	125	100	0	0.0345	0.0795	−0.0489	0.0345	−0.0489
#26	124	55	28	0.0328	0.0216	0.0115	0.0328	0.0115
#27	1245	100	0	0.0436	0.0811	−0.0407	0.0436	−0.0407
#28	123	49	23	0.0327	0.0192	0.0138	0.0327	0.0138
#29	1235	100	0	0.0399	0.0807	−0.0443	0.0399	−0.0443
#30	1234	84	10	0.0325	0.0437	−0.0117	0.0325	−0.0117
#31	12345	100	0	0.0482	0.0786	−0.0331	0.0482	−0.0331
	**Total**	2278	138					
	**%**	73.48	4.45					

**Table A4:** Scenario S4, 6.000 rows, 1:2 ratio of positive/negative values for target variable, 100 subsamples per combination, and the gain ratio attribute evaluations of the five best variables are 0.122, 0.076, 0.02, 0.015, and 0.013 (from most to least relevant).

Comb.	Antecedents	J48 wins	I wins	$\gamma^{X}\left(J48\right)$	Γ(J48)	$\gamma^{X}\left(I\right)$	$\gamma^{V}\left(J48\right)$	$\gamma^{V}\left(I\right)$
#1	5	10	19	0.2652	−0.0004	0.2659	0.0203	0.0212
#2	4	0	0	0.2500	0.0000	0.2500	0.0000	0.0000
#3	45	13	20	0.2653	−0.0004	0.2658	0.0204	0.0211
#4	3	0	0	0.2500	0.0000	0.2500	0.0000	0.0000
#5	35	28	18	0.2651	−0.0003	0.2655	0.0202	0.0207
#6	34	0	0	0.2500	0.0000	0.2500	0.0000	0.0000
#7	345	30	20	0.2651	−0.0002	0.2655	0.0201	0.0206
#8	2	0	0	0.2686	0.0000	0.2686	0.0248	0.0248
#9	25	23	76	0.2686	−0.0022	0.2720	0.0248	0.0293
#10	24	0	38	0.2692	−0.0002	0.2695	0.0256	0.0260
#11	245	21	78	0.2691	−0.0025	0.2728	0.0254	0.0305
#12	23	76	8	0.2686	0.0004	0.2684	0.0248	0.0245
#13	235	23	76	0.2685	−0.0020	0.2715	0.0247	0.0287
#14	231	46	40	0.2692	0.0000	0.2692	0.0256	0.0256
#15	2345	24	76	0.2690	−0.0022	0.2723	0.0254	0.0298
#16	1	0	47	0.2501	−0.0002	0.2505	0.0001	0.0006
#17	15	8	28	0.2653	−0.0004	0.2660	0.0204	0.0213
#18	14	0	47	0.2501	−0.0002	0.2505	0.0001	0.0006
#19	145	11	30	0.2653	−0.0004	0.2659	0.0204	0.0212
#20	13	0	47	0.2501	−0.0002	0.2505	0.0001	0.0006
#21	135	24	25	0.2651	−0.0003	0.2656	0.0202	0.0208
#22	134	0	47	0.2501	−0.0002	0.2505	0.0001	0.0006
#23	1345	27	27	0.2651	−0.0003	0.2656	0.0202	0.0207
#24	12	0	47	0.2687	−0.0002	0.2691	0.0250	0.0254
#25	125	21	76	0.2686	−0.0023	0.2721	0.0247	0.0295
#26	124	0	69	0.2693	−0.0004	0.2699	0.0257	0.0266
#27	1245	18	80	0.2689	−0.0026	0.2729	0.0253	0.0306
#28	123	41	47	0.2687	−0.0001	0.2688	0.0250	0.0251
#29	1235	22	76	0.2685	−0.0020	0.2716	0.0247	0.0288
#30	1234	24	69	0.2693	−0.0002	0.2697	0.0257	0.0262
#31	12345	22	77	0.2691	−0.0022	0.2724	0.0254	0.0299
	**Total**	512	1308					
	**%**	16.52	42.19					

**Table A5:** Scenario S5, between 7820 and 7940 rows, 1:3 ratio of positive/negative values for target variable, 100 subsamples per combination, and the gain ratio attribute evaluations of the five best variables are 0.102, 0.134, 0.016, 0.013, and 0.010 (from most to least relevant).

Comb.	Antecedents	J48 wins	I wins	$\gamma^{X}\left(J48\right)$	Γ(J48)	$\gamma^{X}\left(I\right)$	$\gamma^{V}\left(J48\right)$	$\gamma^{V}\left(I\right)$
#1	5	59	41	0.3336	0.0013	0.3327	0.0191	0.0179
#2	4	81	0	0.3339	0.0016	0.3328	0.0181	0.0165
#3	45	59	41	0.3234	0.0012	0.3226	−0.0003	−0.0007
#4	3	81	0	0.3288	0.0016	0.3277	0.0090	0.0074
#5	35	59	41	0.3209	0.0012	0.3201	−0.0022	−0.0026
#6	34	81	0	0.3187	0.0016	0.3176	−0.0037	−0.0043
#7	345	59	41	0.3310	0.0012	0.3302	0.0141	0.0129
#8	2	73	10	0.3187	0.0012	0.3179	−0.0039	−0.0043
#9	25	59	41	0.3234	0.0012	0.3226	−0.0003	−0.0007
#10	24	73	10	0.3338	0.0012	0.3330	0.0190	0.0178
#11	245	59	41	0.3209	0.0012	0.3201	−0.0020	−0.0024
#12	23	74	9	0.3263	0.0012	0.3254	0.0041	0.0028
#13	235	59	41	0.3109	0.0012	0.3101	−0.0087	−0.0091
#14	231	74	9	0.3313	0.0013	0.3305	0.0146	0.0133
#15	2345	59	41	0.3234	0.0012	0.3226	−0.0006	−0.0010
#16	1	80	1	0.3462	0.0015	0.3452	0.0377	0.0363
#17	15	89	10	0.3331	0.0023	0.3316	0.0112	0.0089
#18	14	93	1	0.3438	0.0020	0.3425	0.0319	0.0300
#19	145	89	9	0.3384	0.0026	0.3366	0.0220	0.0194
#20	13	75	4	0.3424	0.0014	0.3414	0.0291	0.0277
#21	135	93	5	0.3397	0.0026	0.3380	0.0239	0.0214
#22	134	89	4	0.3246	0.0016	0.3235	−0.0018	−0.0024
#23	1345	95	3	0.3398	0.0027	0.3380	0.0240	0.0214
#24	12	74	9	0.3541	0.0013	0.3532	0.0524	0.0512
#25	125	89	8	0.3232	0.0024	0.3215	−0.0026	−0.0034
#26	124	84	9	0.3336	0.0015	0.3326	0.0124	0.0109
#27	1245	89	8	0.3308	0.0026	0.3290	0.0070	0.0044
#28	123	70	13	0.3347	0.0010	0.3341	0.0141	0.0131
#29	1235	91	5	0.3346	0.0025	0.3330	0.0144	0.0119
#30	1234	81	11	0.3398	0.0013	0.3390	0.0236	0.0223
#31	12345	94	4	0.3447	0.0025	0.3431	0.0343	0.0319
	**Total**	2384	470					
	**%**	76.90	15.16					
